# Breast cancer care during a pandemic: an opportune time for cryoablation?

**DOI:** 10.1007/s10549-020-05724-0

**Published:** 2020-06-11

**Authors:** Dennis R. Holmes

**Affiliations:** 1 John Wayne Cancer Institute, Santa Monica, CA USA; 2 Adventist Health Glendale, Glendale, CA USA

**Keywords:** Cryoablation, Cryosurgery, Ablation, COVID-19, Pandemic, Coronavirus

## Abstract

**Purpose:**

Cryoablation is a minimally-invasive percutaneous procedure that is capable of reducing the psychosocial burden of surgical delay while also decreasing the morbidity of breast cancer therapy. The purpose of this editorial is to discuss the potential role of cryoablation for reducing the psychosocial burden of surgical delay during the COVID-19 pandemic by expediting the management of breast cancer while also lessening demand on limited healthcare resources.

**Methods:**

This editorial critiques current expert opinion recommendations that aim to reduce viral transmission and preserve healthcare resources during the COVID-19 pandemic by advocating delay of elective breast cancer surgery.

**Results:**

The editorial summarizes the current state of the evidence that supports the selective use of cryoablation as a definite or stopgap measure in the management of breast cancer during the COVID-19 pandemic or when healthcare resources are limited.

**Conclusions:**

As an office-based procedure performed under local anesthesia, cryoablation eliminates the need for operating room personnel and equipment while also reducing the psychosocial impact of delayed breast cancer surgery. By reducing the number of patient and healthcare provider interactions, cryoablation not only decreases the risk of viral transmission but also the need for personal protective devices during resource-limited times.

## Introduction

Unprecedented times call for novel solutions. The COVID-19 pandemic has placed tremendous burden on healthcare personnel, equipment, and supplies. Social distancing and other efforts to control spread of COVID-19 have led directly to the introduction of “pragmatic” policies that have temporarily but dramatically altered all healthcare in United States and the rest of the world. With regards to breast cancer, many prominent organizations such as The American Society of Breast Surgeons (ASBrS), the American College of Radiology (ACR), and the American Cancer Society (ACS) have recommended immediate postponement of routine mammography, ultrasound, and breast MRI in asymptomatic women for 6–12 months or until after the pandemic is over [1, 2]. It is well known that breast cancer screening is one of most important factors contributing to modern improvements in breast cancer survival. While allowance is made for the ongoing evaluation of patients with symptomatic breast problems, the recent closure or curtailment of work-hours of many breast imaging centers has made it increasingly challenging for women to complete assessments of existing breast problems.

A key driver of the moratorium on routine breast cancer screening is the widely applied hospital policy to postpone elective surgical procedures to preserve limited hospital resources and to minimize viral exposure during the pandemic. This policy has been endorsed by the American College of Surgeons (ACOS) and by an expert opinion Special Communication of the ASBrS, National Accreditation Program for Breast Centers, National Comprehensive Cancer Network, Commission on Cancer, and ACR which recommend at least a 1.5–4 month delay of surgery for most conditions that are not immediately life-threatening (designated Surgical Priority B and C), which include nearly every breast diagnosis, including cancer [1, 3]. In “Recommendations for Prioritization, Treatment and Triage of Breast Cancer Patients During the COVID-19 Pandemic,” the authors advocate expedited breast surgery only for breast conditions that are immediately life-threatening: abscesses and bleeding complications requiring surgical management (Surgical Priority A), prevention or management of wounds with compromised blood supply (Surgical Priority B1), and surgical management of women completing pre-operative chemotherapy for high-risk breast cancer (triple-negative or HER2/neu-positive breast cancer or breast cancers that show continued growth despite pre-operative systemic therapy) (Surgical Priority B1).

In lieu of surgery, the ACOS and the Special Communication recommend pre-operative endocrine therapy 6–12 months for most women with estrogen-sensitive breast cancer as a way of reducing cancer extent or preventing tumor growth (Surgical Priority B3, C1). For example, for women with stage I, node-negative, estrogen-sensitive, HER2/neu-negative breast cancer, the Special Communication recommends endocrine therapy while withholding surgery until after the pandemic has ended (Surgical Priority C1), which directly impacts 70% of women diagnosed with breast cancer in the modern era. This decision is further complicated by compliance concerns due to the side effects many woman experience with endocrine therapy. Women requiring or considering mastectomy are advised to forego breast reconstruction altogether or limit breast reconstruction options to implant-based reconstructions instead of tissue flap reconstruction (Surgical Priority C2) to avoid extended hospitalizations.

Although short surgical delays are unlikely to diminish patient breast cancer survival, an unfortunate consequence of these policies is the tremendous amount of anxiety that follows a patient’s consent to postpone surgery for several months. Anxiety may be created by the possibility of delay resulting in tumor growth or spread and may also be heightened by uncertainty about how long the pandemic will last. Will the pandemic be followed by another surge of viral infections that might further delay the scheduling of elective surgery nationally, regionally, or locally? When the pandemic ends, will the national backlog of elective surgical procedures impede the scheduling of breast cancer operations, and what will determine the order of patients in the queue awaiting surgery—date of diagnosis, tumor stage or biology, patient age, level of insurance, etc.? Will hospitals that have been financially strapped by the lack of elective surgery remain operational and sufficiently staffed to handle the backlog of elective cases? Assuming regional variations in access to surgery, will patients have the freedom of movement, financial means, and insurance coverage to travel from one location to another for breast cancer procedures that might not be available in their home community?

What distinguishes this pragmatic policy from the traditional use of pre-operative endocrine therapy is that neoadjuvant endocrine therapy is most often administered for a period of 6–9 months to increase the odds of lumpectomy in a patient who would otherwise require mastectomy due to large tumor size. Hence, the benefit of improved surgical outcomes helps to ease the anxiety of leaving the tumor in place for an extra 6–9 months. However, from an oncological and philosophical perspective, it’s difficult to rationalize delaying definitive treatment in patients with early stage breast cancer when the potential for surgical benefit is marginal. For example, a woman who is already a candidate for lumpectomy is unlikely to receive sufficient tumor response to neoadjuvant endocrine therapy to eliminate the need for surgery and will still ultimately require lumpectomy. Furthermore, there are insufficient data supporting the efficacy and safety of pre-operative endocrine therapy in pre-menopausal women [4].

Completely missing from the official discourse about the pragmatic management of breast cancer is percutaneous breast tumor ablation, specifically cryoablation or tumor freezing. Although cryoablation has yet to be established as a standard-of-care procedure for the management of breast cancer, unconventional times call for unconventional measures, just like the current moratoria on breast cancer screening and elective breast surgery that the co-authors of the Special Communication acknowledge are based on expert opinion and only limited prospective experience. By these standards, cryoablation warrants special consideration as a pragmatic, non-operative, resource-saving strategy for managing breast cancer.

Cryoablation has emerged as a minimally-invasive alternative to breast cancer surgery that reduces morbidity along with the psychosocial and cosmetic impact of breast cancer therapy. As an outpatient, office-based procedure performed under local anesthesia, cryoablation also reduces burden on the healthcare system by eliminating the need for an operating theater, an anesthesiologist and surgical team and decreasing the number of requisite personal protective devices (e.g., masks, face shields, and surgical gowns)—all important considerations in a pandemic where such resources are at a premium. Using a needle-like, handheld cryoprobe and liquid-nitrogen, tumors are typically ablated in two freeze–thaw cycles achieving a core temperature of – 180 °C. Breast masses ≦ 2 cm can be cryoablated in as little as 30–45 min compared to 2–3 h for lumpectomy or mastectomy. Patients are spared the cost, discomfort, and potential complications of general anesthesia and surgical resection. Skin injury is prevented by repeated injection of saline under the dermis to maintain separation between the skin and the underlying frozen mass. Short and long-term cosmesis and patient satisfaction would likely be superior since the incision is small (~ 3 mm) and there is no need for removal of breast tissue. The procedure is essentially painless due to the analgesic effect of the tumor freezing process, and post-procedure narcotic requirements are nearly nil. Most patients are able to return to non-strenuous activities the very next day.

Although surgical resection offers the advantage of complete tumor removal and margin assessment, apart from excess resource utilization, a major drawback of surgical resection is the cosmetic and functional impairment of the breast resulting from volume changes, scar formation, nipple displacement, sensation changes, and skin/scar retraction. Since cosmesis is a primary goal of breast conservation, and the major determinant of cosmesis is resection volume, cryoablation has the potential to expand the options for breast conservation by avoiding tumor resection altogether thereby reducing the need for more resource-intensive procedures like mastectomy, breast reconstruction, oncoplastic surgery, and contralateral breast symmetry procedures. Thus, cryoablation with a 1–2 cm ablation margin around the cancer might be the optimal compromise that balances the goals of early detection and treatment of breast cancer with the desire for a less invasive, less morbid, less resource-intensive approach to managing breast cancer. Unlike the average hospital outpatient surgical procedure which typically involves 15–20 patient-hospital personnel interactions, cryoablation achieves social distancing by limiting patient to 2–3 healthcare personnel interactions for the typical office-based procedure.

Breast cancer cryoablation builds on the significant body of preliminary data and historical experience in the use of cryoablation for the treatment of benign and malignant breast tumors, including multiple patient series that identified tumor characteristics most conducive to successful ablation [5]. The largest published series was the American College of Surgeons Oncology Group (ACOSOG) Z1072 trial, a multicenter phase II study which sought to determine the rate of successful ablation in 99 women with early stage breast cancer managed initially with percutaneous cryoablation followed four weeks later by lumpectomy or mastectomy [6]. ACOSOG Z1072 was limited to subjects with invasive ductal carcinomas measuring ≦ 2 cm and no extensive intraductal component, of which complete tumor resection was documented in 100% of tumors < 1 cm and 92% of lesions ≦ 2 cm when accounting for tumor multifocality. The findings of ACOSOG Z1072 influenced the design of the FROST Trial (currently enrolling, www.clinicaltrial.gov, #NCT011992250) and the Ice3 Trial (active, non-enrolling, www.clinicaltrial.gov, #NCT02200705), two U.S. non-randomized trials examining the outcome of a similar cohort of women treated with cryoablation without subsequent surgical removal. Interim results of both trials presented at the 2019 annual meeting of the ASBrS demonstrated 1.1% and 1.4% local recurrence rates with 1 year short-term follow-up. In Japan, a clinical trial at Kameda Medical Center (Kamogawa, Chiba, Japan) undertook a prospective investigation of cryoablation in a cohort of women with clinically node-negative, luminal A invasive ductal carcinomas measuring ≦ 1.5 cm managed with cryoablation, sentinel node biopsy, endocrine therapy, and whole breast radiotherapy without subsequent surgical removal. Although results of this ongoing study have yet to be published in manuscript form, an oral presentation at the 2019 ASBrS meeting reported a 0.98% local recurrence rate among 304 subjects of mean age 57 (31–83 years) with median follow-up of 6 years, which provides confidence in the ability of cryoablation combined with adjuvant therapy to maintain long-term local control in a well-selected patient population. As with breast conserving surgery, there remains a potential for local recurrence within or adjacent to the cryoablation site. The fact that unrecognized, clinically occult, microscopic disease might exist in the perimeter of the cryoablated tumor is why radiotherapy and/or adjuvant systemic therapy remain important in the multidisciplinary management of most patients treated with cryoablation. The same is also true for most patients treated with lumpectomy.

Besides cryoablation, several other methods of percutaneous ablation are currently under investigation, including MRI-guided radiofrequency ablation; ultrasound or MRI-guided high-intensity focused ultrasound; stereotactic, MRI, or ultrasound-guided LASER thermotherapy; and ultrasound-guided microwave ablation [5]. However, data supporting alternate percutaneous ablation techniques are largely limited to small feasibility studies involving ablation followed by lumpectomy or mastectomy, without longer term follow-up of unresected tumors. Other limitations include the necessity to perform these procedures in the radiology department as well as the requirement for anesthesiology support for intravenous sedation which is generally required for pain control during these heat-generating ablation procedures.

In the context of percutaneous management of breast cancer, opportunities also exist for the non-operative management of the axilla. Since 2016, The Society of Surgical Oncology’s “Choosing Wisely” Campaign, an initiative of the American Board of Internal Medicine, has encouraged surgeons to avoid “routine sentinel node biopsy in clinically node-negative women ≥ 70 years of age with hormone receptor-positive, HER2/neu-negative invasive breast cancer [7].” This pragmatic recommendation is validated by the low estimated risk of axillary metastasis in this cohort as well as the low rate of axillary recurrence when axillary surgery is omitted among these patients. Whereas selective use of sentinel node biopsy is now widely accepted in women ≥ 70, withholding sentinel node biopsy is more controversial in younger women for whom the status of the axilla has remained a core factor in assessing the need for chemotherapy. However, the primacy of the axillary nodal stage as a prognostic and predictive factor has been gradually eroded by growing confidence in the prognostic and predictive ability of tumor genomic assays as a tool for assessing the benefit of chemotherapy, regardless of patient age or tumor size. For example, tumor gene expression profiling is now capable of identifying genomically low-risk patients that benefit little from chemotherapy despite the presence of high-risk clinical features, including positive axillary nodes, further reducing the need for surgical axillary staging and the use of related healthcare resources.

Since cryoablation intentionally eradicates tumor histology, it is critical to confirm that the diagnostic needle biopsy provided sufficient tissue samples for histological and genomic assays. Otherwise, additional tumor biopsies should be obtained immediately preceding cryoablation to provide tissue for determination of tumor prognosis and selection of adjuvant therapy.

## Pragmatic clinical applications of cryoablation

Although there is a paucity of high-level data supporting the widespread use of cryoablation for the management of breast cancer, there are considerable preliminary and anecdotal data in surgical oncology in general and breast oncology in particular to warrant consideration of cryoablation as a patient-centered strategy for managing breast cancer when patient consent or resource utilization restricts the treatment options. Table 1 identifies several clinical scenarios for which cryoablation might be considered peri-pandemic in the context of shared decision-making between a patient and her multidisciplinary oncology team. “Definitive therapy” refers to cryoablation of the targeted site without anticipated need for subsequent surgical removal. “Stopgap therapy” refers to the use of cryoablation as a temporary solution prior to anticipated surgical resection. It is not the intent of this discussion to promote cryoablation as the preferred solution for each of these clinical scenarios but rather to acknowledge the potential for cryoablation to provide a definitive or stopgap solution in selected patients who are anxious about delaying surgery. However, cryoablation can also play a role in the management of selected patients who refuse traditional surgery.

**Table 1:** Pragmatic applications of cryoablation

		“Special Communication” surgical priority^a^	Cryoablation as definitive therapy	Cryoablation as stopgap therapy
A	Stage I, clinically node-negative invasive breast cancer	C1	Consider	Possibly Consider
B	Stage I & II, clinically node-negative invasive breast cancer	B3, C1	Consider	Consider
C	Stage III, clinically node-negative or node-positive invasive breast cancer	B2, B3	Possibly Consider	Consider
D	Stage 0, ductal carcinoma in situ	C1, C2	Consider	Consider
E	Locally recurrent invasive breast cancer or ductal carcinoma in situ	B3	Consider	Consider
F	Management of the breast primary in Stage IV breast cancer	*	Consider	

B1 = candidate for expedited surgery

B2 = candidate for expedited surgery or 6–12 month surgical delay

B3 = surgical delay recommended until after pandemic

C1, C2 = surgical delay recommended until after pandemic

*Undesignated

^a^COVID-19 Pandemic Breast Cancer Consortium Priority Category

## Stage I, clinically node-negative invasive breast cancer

This is the clinical scenario for which data are most compelling regarding the use of cryoablation. Patients with stage I breast cancer may undergo cryoablation of the primary breast lesion, with delayed resection of the cryoablation site reserved for patients with extensive ductal carcinoma in situ and/or for those requiring surgical axillary staging, e.g., women under 70 years of age or those with hormone receptor-negative, HER2/neu-positive, or genomically high-risk disease. Adjuvant systemic therapy would be guided by the tumor genomic profile and risk of distant recurrence. Women age 70 years and older with hormone receptor-positive disease may forego adjuvant radiotherapy altogether if they agree to take endocrine therapy. For other women, hypofractionated radiotherapy may be initiated when permitted by available resources. Although adjuvant radiotherapy is standard of care for women under age 70, the volume of breast tissue ablated for a 1 cm invasive breast cancer exceeds the volume of tissue resected and irradiated for the typical 1 cm tumor treated with accelerated partial breast radiotherapy, assuming lumpectomy margins with “no tumor on ink.” With this in mind, selected candidates for lumpectomy and APBI may potentially undergo cryoablation without radiotherapy if the area of ablated tissue exceeds the volume that would have been treated with partial breast radiotherapy.

It has been suggested that cryoablation should be initiated only if a tumor proves to be refractory to endocrine therapy due to tumor biology (e.g., low level of estrogen expression) or patient medication non-compliance. However, unlike endocrine therapy which will nearly always requires subsequent surgical resection, cryoablation is capable of completely eliminating the need for breast surgery for a large proportion of women with stage I breast cancer. Therefore, cryoablation might prove to be a more appealing initial therapy wherever cryoablation is available.

## Stage I & II, clinically node-negative invasive breast cancer

Extrapolating from patients with low-risk stage I disease, patients with T2, low-risk, node-negative or T1, high-risk, node-negative breast cancer may receive upfront cryoablation for management of the breast primary combined with adjuvant endocrine therapy if hormone receptor-positive. Sentinel node biopsy with or without resection of the primary tumor may be performed when conveniently scheduled. Endocrine therapy may be initiated immediately after cryoablation followed by adjuvant chemotherapy (if indicated) and/or adjuvant radiotherapy. Surgical resection of the cryoablation site can be performed at a later date if follow-up imaging suggests the presence of residual or recurrent disease.

## Stage III, clinically node-negative or node-positive invasive breast cancer

Drawing on experience from liver ablation, patients with large T2 or T3 breast tumors may undergo cryoablation using multiple overlapping cryoablation zones in a single session to ablate the primary tumor plus a surrounding margin of normal tissue [8, 9]. Cryoablation might eliminate the need for surgery in patients refusing breast surgery or provide temporary control of tumor growth until the patient is able to conveniently undergo mastectomy with or without reconstruction. For patients seeking to avoid implant-based reconstruction, cryoablation can control the primary tumor site until the patient is able to undergo tissue flap reconstruction in a single stage post-pandemic. Unlike liver ablation where high blood flow in large central blood vessels limits the freezing effect of cryoablation on surrounding tissue, the complete absence of large blood vessels in the breast gland makes invasive breast cancer an ideal candidate for cryoablation. Ultimately, the need for drug therapy (e.g., chemotherapy, immunotherapy, endocrine therapy and/or checkpoint-inhibitors), post-mastectomy radiotherapy, or post-cryoablation radiotherapy should be guided by the anatomic tumor extent and/or tumor biology.

## Stage 0, ductal carcinoma in situ

The management of ductal carcinoma in situ (DCIS) with cryoablation is in keeping with the overall trend to managing DCIS non-operatively, such as with endocrine therapy or surveillance alone in selected patients [10]. However, a recent analysis of the impact on pre-operative delays in patients with DCIS revealed a statistically significant (7.4%) increased relative risk of death for every 30-day interval increase, which shows the potential hazard of simply postponing the management of DCIS until after the pandemic (Priority C) [11]. Indirect evidence supporting the efficacy of cryoablation in the management of DCIS may be derived from studies revealing no residual invasive cancer or DCIS in tissue samples obtained from needle biopsies of cryoablated tumors. As a primarily office-based, ultrasound-guided breast procedure, the exclusion of pure DCIS from recent cryoablation trials was largely a consequence of the inability of ultrasound to reliably visualize and target DCIS for ultrasound-guided cryoablation. However, if a focal area of DCIS could be converted to an ultrasound-visible target by strategic insertion of one or more ultrasound-visible biopsy site markers, then even DCIS could be made suitable for ultrasound-guided cryoablation.

## Locally recurrent invasive or non-invasive breast cancer

Similar to primary breast cancer, a localized recurrence of invasive breast cancer or DCIS following prior breast conserving therapy or cryoablation may be managed with cryoablation if pre-treatment imaging confirms the presence of a discrete. Placement of an ultrasound-visible biopsy site marker may be required to facilitate cryoablation.

## Management of the breast primary in Stage IV breast cancer

While the debate continues regarding the benefit of lumpectomy or mastectomy in the setting of stage IV breast cancer, cryoablation may be offered as a low-morbidity strategy to control the primary lesion while systemic therapy and possibly radiotherapy are administered to control the metastatic site(s) [12, 13]. Although beyond the scope of this paper, there is also a role for cryoablation in the management of metastatic lesions.

## Conclusion

The COVID-19 pandemic has placed tremendous burden on healthcare personnel, equipment, and supplies. “Pragmatic” national policies have established a moratorium on routine breast cancer screening and elective breast surgical procedures in an effort to preserve limited hospital resources and limit transmission of COVID-19 between patients and healthcare personnel. Although short-term surgical delay is considered unlikely to diminish breast cancer survival, the moratorium on elective breast surgery has the capacity to greatly increase the psychosocial burden of a breast cancer diagnosis. Among our many responsibilities as oncologists is to manage our patients’ malignancy as well as their anxiety about their diagnosis, treatment, and prognosis. Expedient breast cancer screening and treatment recommendations make achievement of these goals more challenging.

Breast tumor cryoablation is an outpatient, minimally-invasive percutaneous procedure that is already emerging as a substitute for surgery for early stage invasive breast cancer. It can serve as a stand-alone therapy for some patients and a temporizing measure for others. In addition to reducing the morbidity, psychosocial and cosmetic impact of breast cancer therapy, this office-based procedure performed under local anesthesia also reduces the burden on the healthcare system by eliminating the need for an operating theater, an anesthesiologist and surgical team, and many personal protective devices—all resource-saving measures. Cryoablation can expedite definitive treatment in some patients, minimize the risk of disease progression of the primary tumor site, reduce the anxiety of prolonged surgical delay, and save healthcare resources. As a pragmatic alternative to lumpectomy and mastectomy during this pandemic or the next healthcare crisis, cryoablation may serve a strategic role in the multidisciplinary management of both early and advanced breast cancer when patient consent or resource restrictions limit breast cancer treatment options. Access to cryoablation would be greatly aided by relaxation of current insurance coverage restrictions that now form a barrier to the inclusion of cryoablation in the multidisciplinary management of breast cancer.
